# Structure, Genetics and Worldwide Spread of New Delhi Metallo-β-lactamase (NDM): a threat to public health

**DOI:** 10.1186/s12866-017-1012-8

**Published:** 2017-04-27

**Authors:** Asad U. Khan, Lubna Maryam, Raffaele Zarrilli

**Affiliations:** 1 Medical Microbiology and Molecular Biology Laboratory, Interdisciplinary Biotechnology Unit, Aligarh Muslim University, Aligarh, 202002 India; 2 Department of Public Health, University of Napoli Federico II, Italy, Naples, Italy; 3 CEINGE Biotecnologie Avanzate, Naples, Italy

**Keywords:** Enterobacteriaceae, New Delhi-Metallo-Beta-Lactamases, Carbapenemases, Antibiotic resistance

## Abstract

**Background:**

The emergence of carbapenemase producing bacteria, especially New Delhi metallo-β-lactamase (NDM-1) and its variants*,* worldwide, has raised amajor public health concern. NDM-1 hydrolyzes a wide range of β-lactam antibiotics, including carbapenems, which are the last resort of antibiotics for the treatment of infections caused by resistant strain of bacteria.

**Main body:**

In this review, we have discussed *bla*_NDM-1_variants, its genetic analysis including type of specific mutation, origin of country and spread among several type of bacterial species. Wide members of enterobacteriaceae, most commonly *Escherichia coli*, *Klebsiella pneumoniae*, *Enterobacter cloacae*, and gram-negative non-fermenters *Pseudomonas* spp. and *Acinetobacter baumannii* were found to carry these markers. Moreover, at least seventeen variants of *bla*_NDM-_type gene differing into one or two residues of amino acids at distinct positions have been reported so far among different species of bacteria from different countries. The genetic and structural studies of these variants are important to understand the mechanism of antibiotic hydrolysis as well as to design new molecules with inhibitory activity against antibiotics.

**Conclusion:**

This review provides a comprehensive view of structural differences among NDM-1 variants, which are a driving force behind their spread across the globe.

**Electronic supplementary material:**

The online version of this article (doi:10.1186/s12866-017-1012-8) contains supplementary material, which is available to authorized users.

## Background

Although antibiotics were developed to fight infections, the emergence of new resistant markers, especially New Delhi-metallo-beta-lactamases (NDM-1), hampered the capability of all antibiotics of beta lactam group to treat infections caused by microorganisms carrying such resistances. The possible reason for evolving trends of new markers is mutations [1], which may cause delaying in the discovery of new antibiotics for treatments and hence became a great public threat [2]. The overuse of antibiotics is one of the reasons to cause resistance, due to increase selective pressure in a specific population of bacteria allowing the resistant bacteria to bloom and the susceptible bacteria to pass away.

Enzymes are evolving over a period of time by mutations in response to environmental pressure for increased stability and fitness leading to its functional changes. The activity of an enzyme and its future generations success in response to change conditions due to environmental stress and its improved physiological utility for constant perseverance is determined by these evolutionary drivers. Recent reports on antibiotic resistance has made a clear understanding of evolving status of β-lactamase enzymes, which are key player for antibiotic resistance [3].

In Enterobacteriaceae and other Gram-negative bacteria including *Pseudomonas* and *Acinetobacter* species, production of carbapenemases has become a noteworthy mechanism for broad-spectrum β-lactam resistance [4]. Carbapenemases may be defined as specific beta-lactamases, which hydrolyze carbapenem group of antibiotics. These are involved in acquired resistance and belong to Ambler molecular classes A, B and D [4].

Intestinal carriage of carbapenemase-producing organisms (CPOs) is an important source of its transmission [5]. However, detection of carbapenemase producing Gram-negative bacteria has become a major concern for the hospital settings to control infections. Use of multiplex PCR analyses and DNA microarray have been reported as rapid detection systems. Most prevalent carbapenemases detected by these systems are KPC and OXA serine carbapenemases [6].

A number of new variants of class A carbapenemases (e.g., KPC and GES enzymes), class B metallo-beta-lactamases (e.g., IMP, VIM and NDM metallo-beta-lactamases), and class D carbapenemases (e.g., OXA-23) are emerging over time scale. Moreover, over-expression of class C beta-lactamases, such as CMY-10 and PDC type beta-lactamases, which are weak carbapenemases, can also lead to carbapenem resistance, especially in combination with other resistance mechanisms [7].

**Fig. 1:**
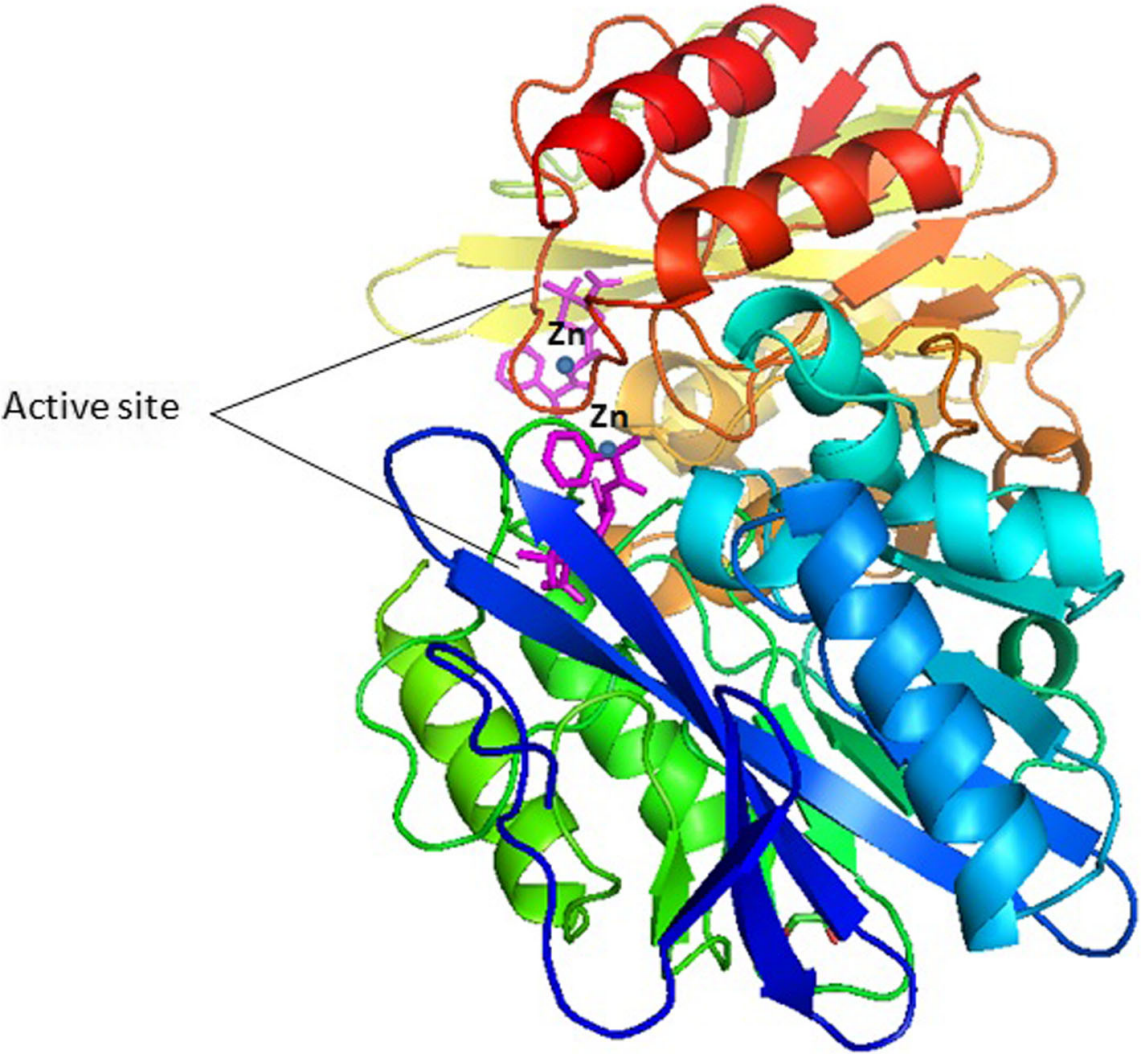
3D structure of NDM-1 protein backbone shown with helices and strands, the two zinc ions at the active sites are shown as blue spheres

Metallo-beta-Lactamases (MBLs) are class B β-lactamases that hydrolyze almost all clinically-available β-lactam antibiotics and feature the distinctive αβ/βα sandwich fold of the metallo-hydrolase/oxidoreductase superfamily. MBLs possess a shallow active-site groove with one or two divalent zinc ions, bordered by flexible loops [8]. In NDM-1 this flexible hairpin loop moves over the zinc ion for hydrolysis and is later removed after the catalysis [9]. The 3D structure of NDM-1 with active site and Zinc molecules is shown in Fig. 1. MBLs are classified into three subclasses (B1, B2 and B3), according to sequence identity and zinc ion dependence, of which the B1 subclass included most clinically significant enzymes. Not many inhibitors have been successfully designed due to the nature of zinc ligands, catalytic mechanisms and the differences among the active site architecture [8]. The evolution of varied and detrimental range of β-lactamases has lost the effectiveness of β-Lactamase inhibitors (BLIs) which could play an important role in combating β-lactam resistance in Gram-negative bacteria [10]. A triple combination of meropenem/piperacillin/tazobactam β-lactams, has been proved as one of the strategies to kill Methicillin-resistant *Staphylococcus aureus* (MRSA) in vitro as well as in a mouse model through a novel synergistic mechanism of action [10].

A bacterium carrying several antibiotic-resistant genes is called multi-resistant bacteria or informally, a “super bacteria” or “super bug”; infections caused by them are difficult to treat [11]. Most probably, a very rare “genetic fusion” is thought to occur between two previously known antibiotic-resistant genes that evolved to a new mutant called NDM-1.The product of the *bla*_NDM-1_ gene is NDM, an enzyme hydrolysing broad range of antibiotics, including the carbapenems, which are considered as last resort of antibiotics. In the last few years, 17 new variants of NDM-1 have been evolved by changing one or two residues at different positions [12–15]. The emergence of bacteria carrying such genes represent a big challenge for physicians to treat infected patients.

### Mechanism of resistance

The expression of β-lactamases, efflux pumps and alteration of porins and penicillin binding proteins (PBPs) are the common mechanism for carbapenem resistance in member of Enterobacteriaceae. Combinations of these mechanisms can cause high levels of resistance to carbapenems in certain bacterial species, such as *K. pneumoniae*, *Pseudomonas aeruginosa* and *A. baumannii*. In *P. aeruginosa* carbapenem resistance is contributed also by the loss of OprD porin leading to decrease in outer membrane permeability, increase in cytoplasmic membrane active efflux pump system, up regulation and alteration in carbapenem hydrolyzing enzymes and penicillin binding proteins [16]. Acquisition of metallo-beta lactamases (MBL), which hydrolyze the carbapenems and all beta lactams except the monobactams, is one of the emerging mechanism of carbapenem resistance [17]. Multi-drug-resistant *Pseudomonas aeruginosa* (MDRPA) infection risk factors include immunocompromised states, prolonged hospitalization and antimicrobial therapy [18]. NDM-1 producing *P. aeruginosa* isolates were detected for the first time in Serbia [19]. In all four *P. aeruginosa* isolates detected, *bla*_NDM-1_ genes were present on 50 kb plasmid (Gene Bank accession numbers JX680682, JX680683, JX680684 and JX680685) [20].The co-expression of *bla*_NDM-1_ and MexAB-OprM efflux pump occurred into a *P. aeruginosa* strain upon single dose of meropenem therapy, thus suggesting that both mechanisms contribute to carbapenem resistance, although the efflux system played the major role [21]. One more example of combinatorial effects in *A. baumannii* harbouring *bla*_NDM_ is the expression of multiple efflux systems and altered membrane permeability [22].

A distinction exists between resistance to carbapenems in Gram-positive cocci and Gram-negative rods. In Gram-positive cocci, carbapenem resistance is typically the result of substitutions in amino acid sequences of PBPs or acquisition/production of a new carbapenem-resistant PBP. Expression of beta-lactamases and efflux pumps, as well as porin loss and alterations in PBP, are all associated with carbapenem resistance in Gram-negative rods [23]. For example, a clinical strain HPC299 *Acinetobacter bereziniae,* harbouring *bla*_NDM-1,_ uses multidrug efflux pumps as its adaptation strategy for survival under different environmental conditions [24]. Carbapenem resistance mechanisms not related to carbapenemase production include increase in efflux pump activity [25] and modifications of outer membrane porin profiles, which regulate access of carbapenems to the cell wall [26].

### Multi-drug resistance by ndm-1 producing bacteria

#### *Background of* NDM-1 *producers*

There are hundreds of commensal strains of *E. coli* bacteria, which are not associated with any infectious diseases. However, emergence of a new mutant strain known as NDM-1 producing *E. coli* has thrown light on the fact that the development of antibiotic resistance among microorganisms can transform commensals into pathogens. Many NDM-1 variants evolved in Enterobacteriaceae, Vibrionaceae and other non-fermenters by single and double amino acid residue substitutions at different positions [27], for e.g., NDM-1 (major variant), NDM-2, NDM-3, NDM-4and NDM-5 (minor variants), reported worldwide [12, 28, 29]. New Delhi metallo-beta lactamase (NDM) produced by bacterial isolates from the Indian subcontinent are the latest carbapenemases, which hydrolyze all beta lactam antibiotics (except aztreonam), including the broad spectrum antibiotic “carbapenems”, thereby causing havoc in hospitals and community [30]. The gene encoding NDM-1 is often carried by plasmids and hence easily moves to other microorganisms via horizontal gene transfer, thereby increasing the probability of emergence of drug resistant strains of pathogenic microorganisms [31].

#### Major healthcare risk of NDM producers

NDM-1 strains are particularly hazardous because: (i) most plasmids detected in these bacteria are transferable and capable of wide rearrangement, suggesting a widespread horizontal transmission and flexibility among bacterial populations; (ii) there is lack of a routine standardized phenotypic test for metallo-beta-lactamase (MBL) detection; (iii) there is consequent probable high prevalence of unrecognized asymptomatic carriers; (iv) there is a lack of available effective antibiotics for the treatment of multi-drug resistant NDM-1 expressing bacteria [31].

NDM-1 producing *E. coli* infects the host by commonly invading sites like, urinary tract, blood, lungs, and wounds, leading to urinary tract infections, septicaemia, pulmonary infections, diarrhoea, peritonitis, device-associated infections and soft tissue infections [12]. These antibiotic resistant bacteria express type IV secretion system as their virulence factor, which allows them to introduce bacterial proteins and enzymes inside the host cell, thereby controlling the host cell metabolism [32]. Mode of transmission of NDM-1 producing strain could either be through cross-contamination during food preparation or via body fluids and may occur in the community or in the hospital setting [33].

#### Worldwide distribution of NDM variants across the globe

Asian continent serves as the major reservoir of NDM producers, with around 58.15% abundance of NDM-1 variant distributed mostly in China and India. Additional file 1: Table S1 shows detailed prevalence of NDM-1 and its variants in different countries worldwide. Europe shows around 16.8% of the total producers, with the maximum spread of NDM-1 variant in Bulgaria, Romania, Poland, France, Italy, Turkey, Germany, Greece, Serbia, London, Ukraine, Croatia, Azerbaijan and Ireland. NDM-4 is also reported to be distributed in European subcontinent in Italy, while NDM-5 and NDM-7 are prevalent in Denmark and France (Additional file 1: Table S1). American continent shows around 10.8% abundance of the total NDM-1 producers as reported globally, of which subcontinent Brazil serves as the major reservoir while Colorado, Mexico city, California, Georgia, Illinois, Paraguay, Pennsylvania, Florida, Argentina, Jamaica, Uruguay and Ecuador are considered as minor pool (Additional file 1: Table S1). Africa carries around 10.8% pool of the total NDM-1 producers scattered globally. African subcontinent, Algeria showed major distribution, whereas Greater Johannesburg Area, KwaZulu-Natal, Libya, Madagascar, Egypt and Tunisia demonstrated low prevalence of these NDM-1 producers. NDM-5 is also reported to be distributed in Algeria (Additional file 1: Table S1). Australia serves as the 1.6% reservoir of the total NDM-1 producers distributed in Brisbane, Perth and New Zealand. Highest distribution of these NDM variants is detected in *K. pneumoniae* and *E. coli* species (Additional file 1: Table S1).

**Table 1:** Genetic variations among the NDM-1 and its variants and its first source of spread

NDM-1 variants	Amino acid(s) substitution	Source organism(s)
NDM-2	Proline 28 to Alanine	*Acinetobacter baumannii*
NDM-3	Aspartate 95 to Asparagine	*Escherichia coli*
NDM-4	Methionine 154 to Leucine	*Escherichia coli*
NDM-5	Valine 88 to Leucine Methionine 154 to Leucine	*Escherichia coli*
NDM-6	Alanine 233 to Valine	*Escherichia coli*
NDM-7	Aspartate 130 to Asparagine Methionine 154 to Leucine	*Escherichia coli*
NDM-8	Aspartate 130 to Glycine Methionine 154 to Leucine	*Escherichia coli*
NDM-9	Glutamic Acid 152 to Lysine	*Klebsiella pneumoniae*
NDM-10	Arginine 32 to Serine, Glycine 36 toAspartic acid, Glycine 69 to serine, Alanine 74 to threonine, Glycine 200 to Arginine	*Klebsiella pneumoniae*
NDM-11	NA	*Escherichia coli*
NDM-12	Glycine 222 to Aspartic acid and Methionine 154 to Leucine	*Escherichia coli*
NDM-13	Aspartic acid 95 to Asparagine and Methionine 154 to Leucine	*Escherichia coli*
NDM-14	Aspartic acid 130 to Glycine	*Acinetobacter lwoffii*
NDM-15	Alanine 233 to valine Methionine 154 to Leucine	*Escherichia coli*
NDM-16	Arginine 264 to Histidine	*Klebsiella pneumoniae*
NDM-17	Valine 88 to Leucine, Methionine 154 to Leucine and Glutamic acid 170 to Lysine	*Escherichia coli*

NDM-1 producers were found resistant to imipenem, meropenem, ertapenem, gentamicin, amikacin, tobramycin, and ciprofloxacin, whereas, isolates were found susceptible to colistin (MICs ≤4 mg/L) and to tigecycline (MICs ≤1 mg/L) [34]. Non-clonal Indian isolates from Chennai had *bla*_NDM-1_ exclusively on plasmids of size ranging from 50 to 350 kb, whereas another clone of *K. pneumoniae* isolated in Haryana was found to have plasmid of predominately either 118 kb or 50 kb, suggesting wide environmental spread of *bla*_NDM-1_ [34]. Plasmid profiling showed that a plasmid of size 50 kb carries *bla*_NDM-1_in Enterobacteriaceae, which were found resistant to almost all antimicrobials except tigecycline and colistin [34].

**Fig. 2:**
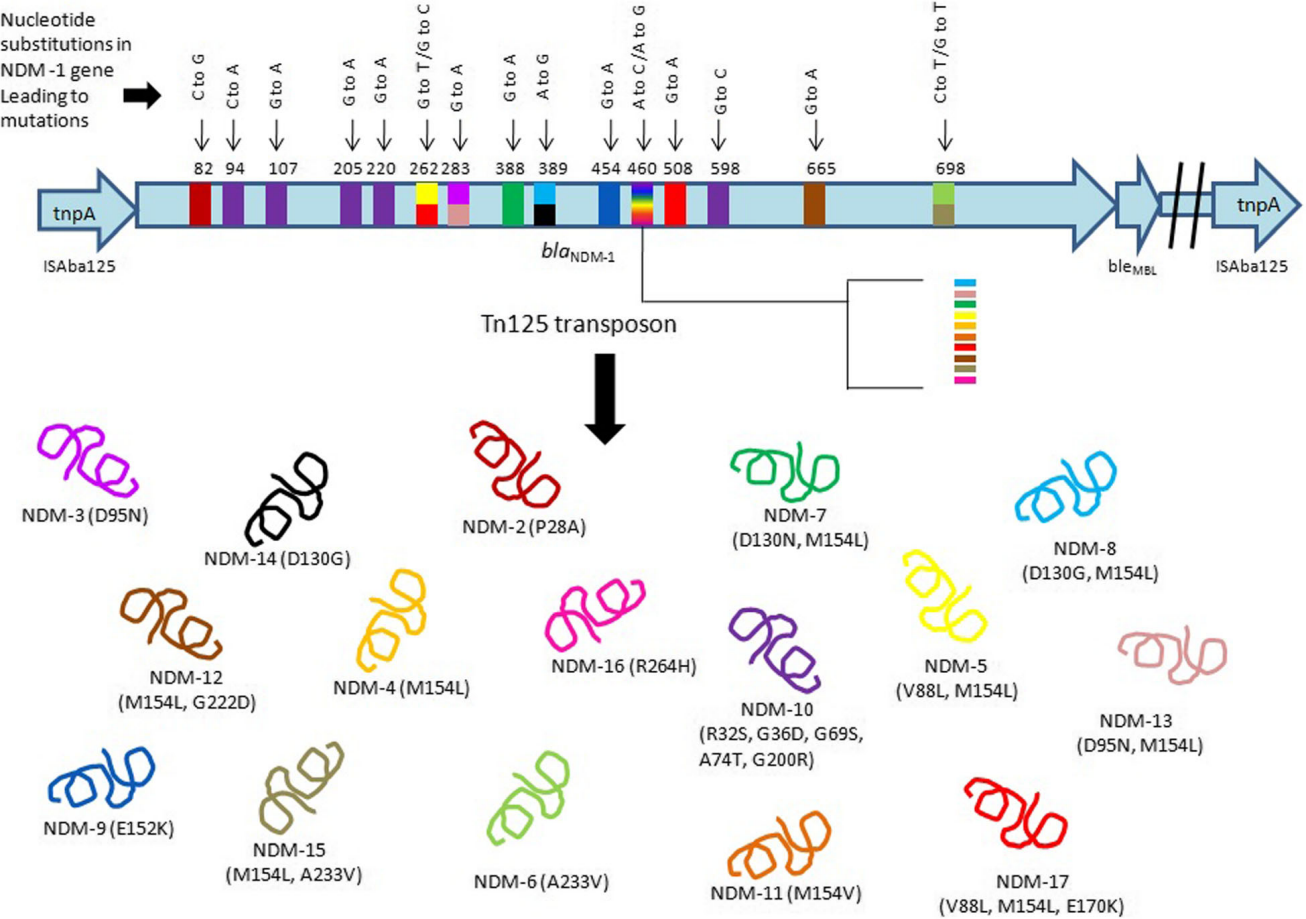
A schematic representation of *bla*_NDM-1_ gene carrying Tn125 transposon, showing the mutations at various nucleotide positions leading to the occurrence of NDM variants. Each unique colour of NDM variant in lower panel showing mutant residues at different position and the same is reflected in gene with the same colour at different position and nucleotide (s)

In Europe, dissemination of NDM-1 has been observed in *A. baumannii* isolates assigned to international clonal lineage I and to the emerging genotypes ST25 and ST85 [35, 36]. The *bla*_NDM-1_ gene was inserted within a Tn125-like transposon which was either chromosomally-located [35] or plasmid-located [35, 36] (Fig. 2).

NDM-producing resistant *E. coli* strains were also found in animal sources [37]. *Acinetobacter lwoffii* carrying *bla*_NDM-1_ gene on plasmid were isolated from chicken rectal swab [38]. The *bla*_NDM_ gene detection in dairy cattle is a matter of concern because it may lead to spread through food chain. Sequence analysis revealed a gene showing 100% homology with *E. coli* (JQ348841.1) *bla*_NDM-5_ gene and 99% homology with *E. coli* (JQ348841.1) *bla*_NDM-4_ in *Pseudomonas aeruginosa* (HF546976.1), *K. pneumoniae* (KC178689.1), *Raoultella ornithinolytica* (JX680686.1), *A. baumannii* (KC404829.1, KC347597.1). Apart from this, NDM-1 producing *Enterobacter cloacae* (EC15) and *K. pneumoniae* (KP12) strains were isolated from two patients with diabetic foot ulcers in 2010 from northern part of India [39].

The origin of NDM-1 started in the year 2008 when the first case of a NDM-1 episode was reported in a Swedish patient previously hospitalized in New Delhi, suffering from a multidrug-resistant *K. pneumoniae*, urinary tract infection [40]. Based on the number of victims affected with NDM-1 strains in various parts of the globe, it has been estimated that the Indian subcontinent is the main reservoir of NDM-1 producers [39], next down the line is United Kingdom. On the other hand, Belgium, China, Japan, France, Austria, Germany, Norway, Hong Kong, Sweden, Netherland, Australia and Canada also serve as the secondary reservoirs of *bla*_NDM_ genes [39] as shown in Additional file 1: Table S1. An average of 1000–1600 patients are admitted daily to the hospitals worldwide as a result of infections due to drug resistant bacteria [41]. It is difficult to predict the rate of spread of the gene encoding NDM- 1, although exchange of the *bla*_NDM-1_ gene among unrelated bacterial isolates have been identified already in *Enterobacteriaceae* and *A. baumannii* [34]. An increase in population exchange at global level and enhanced medical tourism could play a significant role in spreading uncontrolled NDM-1 related resistance worldwide.

#### Genetic and Biochemical analysis of NDM variants

The *bla*_NDM-1_ gene which encodes for the New Delhi metallo-β-lactamase 1 (NDM-1) is commonly found among members of *Enterobacteriaceae* and *Pseudomonas* species [34, 42]. The above bacteria are highly resistant to all antibiotics including carbapenems and aminoglycosides because of co-existence of *rmtF* methylase gene in most of the isolates [43], but susceptible to tigecycline and colistin [34]. However, *bla*_NDM-9_ producing colistin resistant *E. coli* strain was recently discovered in a chicken meat sample in Guangzhou, China [44]. 16S rRNA methyl transferases responsible for high resistance to antibiotics were reported in *bla*_NDM-1_ positive *Pseudomonas aeruginosa* isolates in co-association with *rmtC* and *rmtF* genes on the chromosome [45]. IncR plasmid carrying *bla*_NDM-1_ was also reported in *Citrobacter koseri* acting as a reservoir for multidrug resistance [46]. *bla*_NDM-1_ was associated with different plasmid scaffolds (IncFII, IncL/M, IncN, IncR, IncHIB-M/FIB-M), IncF type being the prevalent one. Genetic structures surrounding *bla*_NDM-1_ showed its association with at least a remnant of ISAba125 at its 5′-end [47]. Tn125 composite transposon in *A. baumannii* has been demonstrated to be responsible for *bla*_NDM-1_ gene dissemination within *Acinetobacter* species and *Enterobacteriaceae* [48]. *ble*_MBL_ gene, which confers resistance to anti-tumor glycopeptide molecule bleomycin, is found downstream of *bla*_NDM-1_ gene [49] (Fig. 2). There has been an exponential increase in resistance among Gram-negative bacteria compared with Gram-positive bacteria [50, 51], while not many new active antibiotics are developed against Gram-negative bacteria [52–54]. Increase in its resistance is mainly due to the presence of mobile elements into conjugative plasmids, which can readily spread through bacterial populations.

The isolates obtained from UK had a more diverse range of plasmid sizes ranging from 80 kb to greater than 500 kb [34]. For example, a RB151 strain was reported to harbour 108 kb plasmid carrying NDM-1 gene on 4.8 Mbp chromosome [55]. *E. coli* Y5 isolate was found to have *bla*_NDM-1_ on chromosome as well [56].The *bla*_NDM-1_ was also carried by more than one plasmid in some isolates. Most of the plasmids carrying *bla*_NDM-1_ shows transmissibility and plasticity enabling them to diversify and spread among bacterial populations with an alarming potential; many of them were of incompatibility A/C types [34], which is not commonly associated group among multidrug-resistant phenotypes. The emergence of new variants of NDM-1 are taking place in India due to widespread use of antibiotics leading to huge selection pressure. Only few antibiotics against Gram-negative bacteria are available and none of them is active against producers of NDM-1 [57]. Large conjugative plasmids are seen to harbour bla_NDM-1_ gene along with determinants of antibiotic resistance [58].

In the United States, *K. pneumoniae* is the most common CRE (carbapenem-resistant *Enterobacteriaceae*) species, resistant to nearly all available antibiotics encountered, typically as a hospital-acquired infection with high mortality and morbidity rate [59, 60]. ATCC BAA-2146 (Kpn2146) a strain of *K. pneumoniae* was the first reported U.S isolate encoding NDM-1 along with additional antibiotic resistance determinants on plasmid of size 140.8 kb.

At least one zinc atom is present on the active site of all metallo-carbapenemases, which facilitates bicyclic β-lactam ring hydrolysis [61]. Metallo-carbapenemases have the ability to hydrolyze commercially available carbapenem resistant β-lactamase inhibitors but show sensitivity to metal ion chelators. Along with carbapenems they can also hydrolyze penicillins and cephalosporins, while the ability to hydrolyze aztreonam is lacking. Hydrolysis occurs when zinc ions on active site interacts with β-lactams giving distinctive inhibition trait by EDTA.

It has been reported that the *bla*_NDM_ is carried by various types of plasmids such as IncA/C, IncF, IncNIncL/M or untypable/IncR, and is rarely found to be chromosomally integrated [62]. Plasmid characterization demonstrated that different mechanism leads to acquisition of NDM gene even if it is located on very closely related plasmids [63]. The sequencing of few plasmids reveals *bla*_NDM_ association with insertion sequences and transposons, which regulate its horizontal gene transfer and *aadB*, *dfrA12*, *bla*_OXA-30_ and *aacA4* additional resistance markers [64]. Tn3000 transposon has been reported to be responsible for *bla*_NDM-1_ dissemination among enterobactericeae [65]. A non-active site residue Trp 93 is found to play role in maintaining the structural integrity of NDM-1, although not being directly involved in recognition and catalysis [66]. Recently, a new plasmid type IncX3 is reported to be responsible for making the spread of NDM gene more effective [67], for example in China IncX3 and InA/C plasmids were reported to be responsible for spread of *bla*_NDM_ genes [68].

Recent studies demonstrated the coexistence of NDM-1 gene along with other resistant genes, such as IMP-1 in *Acinetobacter* species [69], co-expression of NDM-1 and OXA-232 in an *E. coli* isolate was reported from Germany [70], co-production of NDM-5 and MCR-1 in ST648 and ST156 *E.coli* isolates from fowl in China [71]. Coexistence of *bla*_NDM-1_ and *bla*_OXA-48_ carrying plasmids was reported in an isolate of *K. pneumoniae* from China [72]. Similarly, coexistence of *bla*_NDM-1_ and *bla*_OXA-23_ was reported in *A. baumannii* from Nepal [73]. *K. pneumoniae* of type 11 was reported in Greek to be coproducing two metallo beta-lactamses markers, NDM-1 and VIM-1 together [74]. A report on *E. coli* isolate obtained from a patient in Thailand and another report from China, showed the presence of colistin resistant mcr-1 gene along with beta lactamases genes such as NDM-1 [75–77]. In *E. coli* ST471 isolated from Turkey, NDM-1 was found present along with CTX-M-9, TEM, SHV and rmtC [78]. Another coexistence of *bla*_SHV-12_ along with *bla*_NDM-13_ on a self-transferable plasmid of about 54 kb size isolated from *E. coli* in China was reported [79]. Coproduction of NDM-5 along with mcr-1 in China, NDM-7 along with OXA-48 in Spain and NDM-9 along with MCR-1 in Taiwan was reported recently [80–82]. Also, the presence of both NDM-9 and MCR-1 was reported in *Cronobacter sakazakii* and *E. coli* [83].

To date, a number of variants of New Delhi metallo-β-lactamase-1(NDM-1) have been reported. Of these variants, NDM-2 had a substitution of Cysteine to Glycine at position 82, and amino acid being substituted by alanine at position 28 in place of proline, in *A. baumannii* [84] (Table 1). However, 16S RNA methylase and extended-spectrum- β-lactamases were not detected. Moreover, strains carrying *bla*_NDM-2_ lacked detectable plasmids and the *bla*_NDM-2_ was not seen to be transferred by conjugation [84].

Another variant NDM-3 with an amino acid substitution of Aspartate to Asparagine at position 95 was observed in *E. coli* [85] (Table 1). NDM-3 showed similar enzyme activities against β-lactams like those of NDM-1, although slightly lower K_cat_/K_m_ ratios for all the β-lactams tested except for doripenem was seen, which is caused by the lower K_cat_ values of NDM-3 being 19.0 to 47.5% as compared to NDM-1 [86]. In fact, the decreased K_cat_ values and the decrease in hydrolysis rate of all tested β-lactams except for doripenem is due to subsitution of Asn from Asp at position 95. Residue 95 is found to be in α1, located on the surface of the protein [86]. The crystal structure study of NDM-1 revealed that the NDM-1 active site is located at the bottom of a shallow groove being enclosed by two important loops named L3 and L10. However, α1 95th residue was not located in these loops and indirectly may affect the interaction of the substrate with the active site [86]. Among 9 NDM variants, substitutions of amino acids were identified at 7 different positions (28, 88, 95, 130, 152, 154, and 233), but which position(s) plays a critical role in the enzymatic activities, remained unclear. For *bla*_NDM-3_ the genetic context *tnpA-bla*_NDM-3_-*ble*_MBL_-*trpF-dsbC-tnpA-sulI-qacEdeltaI-aadA2-dfrA1*, was present on approximately 250-kb plasmid. The *bla*_NDM-3_ and *bla*_NDM-1_ gene expression in *E. coli* DH5α conferred reduced susceptibility and resistance to all cephalosporins, moxalactam, and carbapenems. *E. coli* expressing NDM-3 showed 2-fold higher MIC of cefpirome than the one expressing NDM-1 in contrast to those of 2-fold lower MIC of cefepime, cefoselis, cefotaxime, cefoxitin, imipenem, meropenem, and penicillin G for NDM-3 than NDM-1. Recombinant NDM-3 and NDM-1 hydrolyzed all tested β-lactams except for aztreonam [86].

NDM-4 variant showed substitution of amino acid from 154th Methionine to Leucine in *E. coli* [87] (Table 1). NDM-4-producing *E. coli* isolate from a North Indian hospital sewage was recently reported by Khan and Parvez [15]. Gene expression of *bla*_NDM-1_ and *bla*_NDM-4_ in *E. coli* TOP10 conferred lower susceptibility or resistance to all β-lactams except aztreonam. However, the MICs of imipenem and ertapenemwere found to be higher for *E. coli* expressing NDM-4 than the one expressing NDM-1, suggesting the role of Leu154 residue in the high carbapenemase activity [87]. NDM-4 β-lactamase hydrolyzed all tested β-lactams except for aztreonam, just similar to other MBLs. Kinetic data showed higher level of hydrolysis of imipenem by NDM-4 than by NDM-1. Similarly, catalytic activity of NDM-4 for meropenem was slightly higher than that of NDM-1. NDM-4 showed higher catalytic efficiencies for cefalotin, ceftazidime, and cefotaxime, as cefepime was less hydrolyzed by NDM-4. Higher K_cat_ values for NDM-4 than NDM-1 for cefalotin and cefotaxime was also observed. *K*_*m*_ values of 72 and 181 μM for NDM-4 and NDM-1 was observed, respectively. NDM-4 showed lower affinity for ceftazidime than NDM-1 [87]. *bla*_NDM-4_ was found on IncF type plasmid in one of the earlier studies [88]. A remnant of insertion sequence IS*Aba125* on upstream of the *bla*_NDM-4_was found previously by PCR mapping during study of genetic structures surrounding the *bla*_NDM-4_ gene [89]. The *ble*_MBL_, a bleomycin resistant gene, was identified downstream of the *bla*_NDM-4_, similar genetic environment has been observed for most of the analyzed NDM-1 positive enterobacterial isolates [88]. PCR-based replicon typing showed that this *bla*_NDM-4_ positive plasmid belongs to the IncFIA incompatibility group. In keeping with this, *bla*_NDM-5_ was also found associated with IncFIA [28, 87].

The substitution of Valine by Leucine at position 88 and Methionine by Leucine at position 154 was found in NDM-5, which was first detected in *E.coli* [28] (Table 1). NDM-5 shows greater hydrolytic activity than NDM-1 toward carbapenems, cefotaxime, cephalotin and ceftazidime [85]. NDM-5 carrying plasmid of size >100 kb reduced susceptibilities of *E. coli* transformants to carbapenems and cephalosporins [28]. Other detected resistance determinants in NDM-5 producing *E. coli* included *dfrA17* and *aadA5* genes, which were found to be located within a class I integron structure, and the 16S rRNA methylase gene, *rmtB*, which was thought to account for aminoglycoside high-level resistance. The effect of NDM-5 on susceptibility of *E. coli* to carbapenems and expanded-spectrum cephalosporins appeared to be greater than that of NDM-1. Sequence analysis of 5′-flanking region of *bla*_NDM-5_ allele revealed presence of partial IS*Aba125*, likely to be derived from *A. baumannii*, which generated a hybrid (−35/−10) promoter as described earlier by Poirel et al. in an NDM-1-producing *E. coli* isolate [90]. NDM-6 showed substitution of Alanine to Valine at 233 position, again first time detected in *E. coli* [12] (Table 1).

Substitutions of Aspartate to Asparagine at position 130 and Methionine to Leucine at position 154were found in NDM-7, identified in *E. coli* ST599 [91] (Table 1). TOP10 cells carrying plasmid harbouring *bla*_NDM-7_ in *E.coli* conferred higher resistance to carbapenems than a plasmid carrying *bla*_NDM-1_ [91]. A recent report demonstrated the role of Leu154 in enhancing carbapenem MICs in NDM-7 producing *E. coli* strain [91]. The *bla*_NDM-7_ gene was found to be located on a self-transferable IncX3 plasmid of 60 kb.

NDM-8 variant having substitutions at positions 130th (Aspartic acid to Glycine) and 154th (Methionine to Leucine) resulted in enzymatic activities against β-lactams similar to those shown by NDM-1 [92]. NDM-9 differing by a single amino acid substitution (E152K) from NDM-1 was recently identified in *K. pneumoniae* ST107 strain from China [93].

NDM-10 was first identified in *K. pneumoniae* isolated from Maharashtra, India and was found to have multiple substitutions at Arginine 32 to Serine, Glycine 36 to Aspartic acid, Glycine 69 to Serine, Alanine 74 to Threonine and Glycine 200 to Arginine [94].

NDM-11 was reported in *E. coli* KnPEc14 strain (Gene Bank KP265939.1).

NDM-12 has two amino acid substitutions at 154th (Methionine to Leucine) and 222th (Glycine to Aspartic acid). It was first identified on plasmid size 160 kb in *E. coli* [95] (Table 1). NDM-12 enzymatic activities were similar to those of NDM-1 against β-lactams, although k_cat_/K_m_ ratios for all β-lactams were tested except doripenem.

NDM-13, a novel New Delhi Metallo-β-lactamase was identified in Nepal from the urine sample of patient showing a carbapenem-resistant *E. coli* infection [96]. It showed substitutions of Asparagine in place of Aspartic acid at position 95 and Leucine in place of Methionine at position 154 (Table 1) and similar enzymatic activity against β-lactams, but higher K_cat_/K_m_ ratios for cefotaxime compared with NDM-1. The *bla*_NDM-13_ gene was located into the chromosome within the genetic environment of *tnpA*-*IS30*-*bla*_NDM-13_-*ble*_MBL_-*trpF*-*dsbC*-*cutA-groES-groL*. Recently, complete sequence of pNDM13-DC33 plasmid harbouring *bla*_NDM-13_ isolated from *E. coli* isolate ST5138 in China, was reported, consisting of a backbone of 33 kb size and encoding an antimicrobial resistance region of 21 kb; *tra, trb* and *pil* transfer functions; *repB* plasmid replication gene and stability partitioning. pNDM13-DC33 plasmid harbouring *bla*_NDM-13_ gene showed high similarity with pNDM-HN380 IncX3 plasmid harbouring *bla*_NDM-1_ gene [79].

NDM-14 was first identified in clinical isolate of *Acinetobacter lwoffii* with substitution of Aspartic acidat 130th position to Glycine [97] (Table 1). NDM-14 showed higher enzymatic activities than NDM-1 towards carbapenem. NDM-14 have higher affinity for meropenem and imipenem than NDM-1, as indicated by the kinetic data [97].

NDM-15 was reported in an *E. coli* strain (Gene Bank KP735848.1). It showed substitution of Alanine to valine at 233th position and Methionine to Leucine at 154th position.

NDM-16 variant showed substitution at 264th position of Arginine to Histidine [98].

**Fig. 3:**
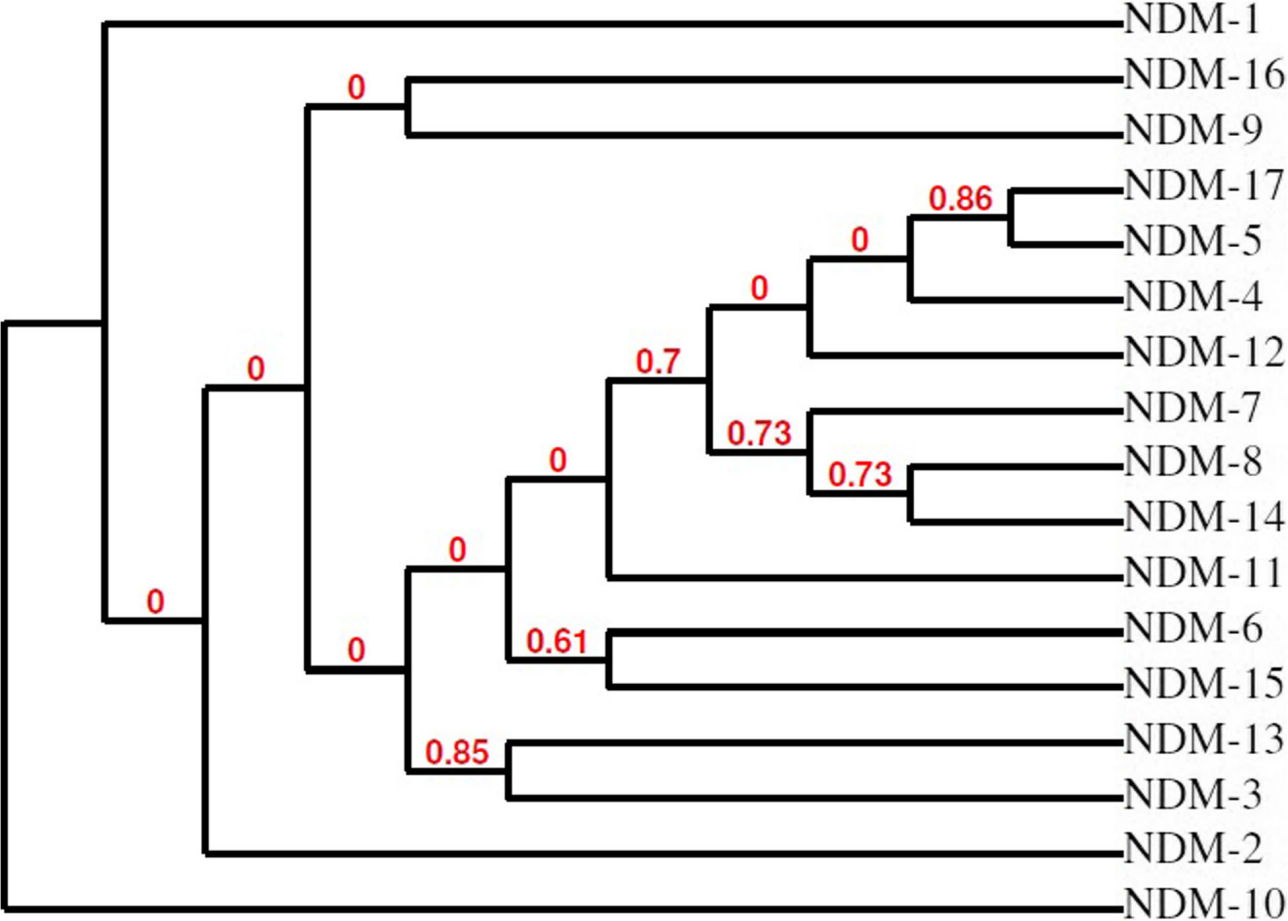
The phylogenetic relationship between protein sequences of NDM variants is shown. The tree construct has been generated using Phylogeny.fr, which used the maximum likelihood method to generate phylogenetic tree [107, 108]

NDM-17 was reported in *E.coli* strain from a chicken in China. It showed amino acid subsitution of valine 88 to leucine, methionine 154 to leucine and glutamic acid 170 to lysine [99]. Schematic representation of mutations on various nucleotides leading to formation of new NDM variant is shown in Fig. 2. Phylogenetic analysis among the protein sequence of NDM variants are represented as cladogram in Fig. 3.

Carbapenem hydrolysing activity was gradually reduced from NDM-7 to NDM-5, NDM-6 and NDM-1. All isolates positive for variants of *bla*_NDM-1_ showed resistance to aminoglycosides with MIC greater than 256 mg/L and MIC range of 2–512 mg/L for different lactams, lactams/lactamase inhibitor combinations [100]. Moreover, these variants showed susceptibility to tigecycline and colistin except for KNKp6a isolate, which showed MIC of 1.5 mg/L to tigecycline [100].

NDM variants were found associated with all other groups of antibiotic resistance enzymes encoding genes i.e. ESBL, carbapenemase, AmpC and rRNAmethylase. In *bla*_NDM_ and its variants, due to genetic co-existence of other antibiotic resistant markers, there is limited options left to treat infections [101].

Recently, an NDM-1 producing *Cedecea lapagei* isolated from a neonate admitted to the paediatric ICU of a north India hospital was reported from our lab [102]. Also, a recent study demonstrated that 11 out of 55 patients with carbapenem-resistant Enterobacteriaceae nosocomial infections in China showed NDM variants as carbapenemase genes [103]. Recently, metabolite aspergillomarasmine A (AMA) which is found in fungi and its natural LLL isomer were identified to be effective inactivators of NDM-1 enzyme both in vivo and in vitro [104]. Also, the combination of levofloxacin and tigecyclinewas recently reported to successfully treat nosocomial pneumonia caused by NDM-1 producing *Raoultella planticola* [105].

## Conclusion

The continual evolution of resistant markers due to the selection pressure and their spread among the bacteria through horizontal gene transfer is one of the alarming threats to the health worker in the hospital settings in order to control infections. NDM-1 and its variants producing bacteria was one of the challenges, which has became even more urgent since the detection of *mcr-1* gene in Chinaand the spread of resistance against colistin has ended all hopes to control infections [77]. Now this is a time to think prudently the ways to check infections from hospital settings and to coordinate globally for surveillance of such resistant markers producing bacteria. Proper infection control guidelines need to be implemented worldwide. Surveillance should also be carried out to identify undetected asymptomatic carriers of carbapenem-resistant bacteria. To discover new drug molecules which could fight with multi-resistant bacteria, Infectious Disease Society of America has launched a “bad bugs need drugs” campaign to promote development of new antibiotics by 2020 [106].

## Additional file

Additional file 1: Table S1. Worldwide distribution of NDM producing bacteria, as per articles available on PubMed database in the time period of Dec 2013 to Feb 2017. (PDF 136 kb)

## Abbreviations

AMA: Aspergillomarasmine A
BLIs: Beta lactamse inhibitors
CPOs: Carbapenem producing organisms
CRE: Carbapenem resistant enterobacteriaceae
ESBL: Extended spectrum beta lactamase
MBLs: Metallo beta lactamses
MDRPA: Muti-drug-resistant pseudomonas aeruginosa
MRSA: Methicillin resistant *Staphylococcus aureus*
NDM: New delhimetallo beta lactamse
PBPs: Penicillin binding proteins
