# Supplementary material for: Structure, Genetics and Worldwide Spread of New Delhi Metallo-β-lactamase (NDM): a threat to public health
Source: BMC Microbiol. 2017 Apr 27;17:101. doi: 10.1186/s12866-017-1012-8 (PMC5408368; doi:10.1186/s12866-017-1012-8)
Supplement: Additional file 1: Table S1. — Worldwide distribution of NDM producing bacteria, as per articles available on PubMed database in the time period of Dec 2013 to Feb 2017. (PDF 136 kb) [file 12866_2017_1012_MOESM1_ESM.pdf]

Table S1. Worldwide distribution of NDM producing bacteria, as per articles available on PubMed database in the time period of Dec 2013 to Feb 2017.

Number	Continent / Country	Variant / Spp	No. of NDM Producing Isolates	Date of Detection	Source / Reference
1.	Asia/Dhaka, Bangladesh	NDM/NA	241	2015 Jun	PMID: 25989320
2.	Asia/Bangladesh	NDM-1/ *Escherichia coli (6), Klebsiella pneumoniae (4), Pantoea spp. (1), Acinetobacter baumannii (1), Enterobacter cloacae (1)*	13	2014 Apr	PMID: 24489109
3.	Asia/Shenyang, China	NDM-5 / *Escherichia coli*	1	2015 Oct	PMID: 26482388
4.	Asia/Changchun, China	NDM-1 / *Acinetobacter lwoffii*	1	2015 Dec	PMID: 26470987
5.	Asia/Southern China	NDM-1 / *A. baumannii*	2	2015 Dec	PMID: 26470986
6.	Asia/Henan Province, China	NDM-1 / *E. cloacae*	8	2015 Oct	PMID: 26452278
7.	Asia/Chengdu, China	NDM-1/*E. coli*	1	2015 Jul	PMID: 26194736
8.	Asia/Guangzhou, China	NDM-1/*Citrobacter freundii, Escherichia coli, Acinetobacter baumannii*	3	2015 Aug	PMID: 26055374
9.	Asia/Beijing, China	NDM-14/ *Acinetobacter lwoffii*	1	2015 Apr	PMID: 25645836
10.	Asia/South China	NDM-1/*K. pneumoniae (3), K. oxytoca (1), E. Cloacae (1), E. hormaechei (1), E. aerogenes (1), and Acinetobacter spp. (2)*	9	2015 Apr	PMID: 25469995
11.	Asia/China	NDM-1/*K. pneumoniae (4), Enterobacter cloacae (1), Enterobacter aerogenes (1) and Citrobacter freundii (1)*	9	2014 Dec	PMID: 25469701
12.	Asia/China	NDM-1/ *Acinetobacter junii and Acinetobacter calcoaceticus*	2	2015 Feb	PMID: 25349061
13.	Asia/China	NDM-1/*Acinetobacter calcoaceticus*	1	2014 Sep	PMID: 25181293
14.	Asia/China	NDM-1/ *Enterobacter cloacae*	1	2016 Jan	PMID: 26787700
15.	Asia/Henan Province, China	NDM-1/*E. coli (6), K. pneumoniae (4), K. oxytoca (1), E. cloacae (3), C. freundii (2)*	16	2014 Aug	PMID: 24777095
16.	Asia/China	NDM-1/*Raoultella planticola, Escherichia coli*	2	2014 Mar	PMID: 24594606
17.	Asia/Beijing, China	NDM-1/*E. coli (1), K. pneumoniae (1), Providencia rettgeri (1), Enterobacter cloacae (1), and Raoultella ornithinolytica (1).*	5	2014 Feb	PMID: 24456600
18.	Asia/Wenzhou, China	NDM-1/ *E. coli*	2	2013 Dec	PMID: 24313961

19.	Asia/Hangzhou, China	NDM-1/ *A. nosocomialis (3), A. pittii (4)*	7	2014 May	PMID: 24306098
20.	Asia/Zhejiang Province, China	NDM-1/ *Salmonella strain*	1	2013 Dec	PMID: 24274898
21.	Asia/ Hong Kong, China	NDM-1/ *E. coli (2), K. pneumoniae (1)*	3	2016 Jan	PMID: 26740321
22.	Asia/ China	NDM-5/ *E. coli*	3	2016 Jan	PMID: 26482388
23.	Asia/ China	NDM-5/ *E. coli*	1	2016 Feb	PMID: 26542305
24.	Asia/China	NDM-7/*E.coli*	5	2016 Apr	PMID: 27216384
25.	Asia/Guangzhou, China	NDM-9/ *E. coli*	1	2016 Jan	PMID: 26842777
26.	Asia/Shanghai, China	NDM-5/*Proteus mirabilis*	1	2016 Mar	PMID: 27065982
27.	Asia/Yunnan, China	NDM-1/ *Klebsiella pneumoniae*	8	2016 Feb	PMID: 26896089
28.	Asia/China	NDM-5/*Klebsiella pneumoniae*	1	2016 Mar	PMID: 26988061
29.	Asia/China	NDM-9/ *E. coli*	1	2016 Mar	PMID: 26842777
30.	Asia/China	NDM-1.*C.werkmanii*	1	2016 Sep	PMID: 27667823
31.	Asia/China	NDM-5/*E.coli*	2	2016 July	PMID: 27406405
32.	Asia/China	NDM-7/*E.coli*	5	2016 July	PMID: 2721638
33.	Asia/Ningxia Provice/China	NDM-1/*Enterobacter cloacae*	8	2017 Jan	PMID: 28197140
34.	Asia/China	NDM-1/*Klebsiella pneumonia*	7	2017 Jan	PMID: 28109845
35.	Asia/China	NDM-3/*S. typhimurium*, NDM-5/*E.coli*	1162	2017 Feb	PMID: 28104504
36.	Asia/China	NDM-1/ *Citrobacter werkmanii*	1	2016 Nov	PMID: 27667823
37.	Asia/China	NDM-4/*E.coli*	1	2016 Nov	PMID: 27876781
38.	Asia/China	NDM-1(36);NDM-5/*S. typhimurium (8)*; NDM-3/ *E.coli (1)*	45	2017 Feb	PMID: 28104504
39.	Asia/Manila,Phiippines	NDM-7/*Klebsiella pneumoniae*	2	2016 Mar	PMID: 27032000
40.	Asia/Czech Republic	NDM-1/*K. Pneumonia*	2	2015 Feb	PMID: 25421477
41.	Asia/Egypt	NDM-1/*Pseudomonas aeruginosa*	1	2014 Dec	PMID: 25449240
42.	Asia/Egypt	NDM-5/ *Escherichia coli*	1	2016 July	PMID: 27173077
43.	Asia/Egypt	*NDM-5/ Escherichia coli*	1	2016 Apr	PMID: 27048740
44.	Asia/Varanasi, India	NDM-1/*P. aeruginosa*	3	2015 Jan	PMID: 25635921
45.	Asia/Kashmir valley, India	NDM-1/*Escherichia coli (2), Klebsiella pneumoniae (2), Citrobacter freundii (3), Acinetobacter spp (1), and Pseudomonas aeruginosa (1)*	9	2014 Nov	PMID: 25579151
46.	Asia/South India	NDM-1/*Pseudomonas aeruginosa*	4	2014 Oct	PMID: 25488450
47.	Asia/West Bengal, India.	NDM-1/*E coli (6), Klebsiella pneumoniae (6), Enterobacter cloacae (3)*	15	2014 Nov	PMID: 25406074
48.	Asia/Chandigarh, India	*NDM-1/Klebsiella pneumoniae*	1	2014 Sep	PMID: 25298566

49.	Asia/Delhi	NDM-1/ *Escherichia coli, Acinetobacter, Klebsiella pneumoniae*	4	2016 Jan	PMID: 26776143
50.	Asia/Punjab, India	NDM/*Acinetobacter calcoaceticus-A. baumannii complex*	32	2014 Oct-Dec	PMID: 25297039
51.	Asia/Bangalore, India	NDM-1/*Acinetobacter baumannii (4), Escherichia coli (5), Klebsiella pneumoniae (8), Providencia rettgerii (8), Enterobacter cloacae (4), Proteus vulgaris (2), Burkholderia cepacia (1), Roultella ornitholytica (1), Pseudomonas putida (1)*	34	2014 Apr	PMID: 24927351
52.	Asia/Kuwait	NDM-1/*Klebsiella pneumoniae*	21	2016 Mar	PMID: 27031521
53.	Asia/Lucknow, India	NDM-1, NDM-5 (2), NDM-6 (8), NDM-7 (3)	57	2014 Jul	PMID: 24831713
54.	Asia/India	NDM-1/*Acinetobacter baumannii*	9	2014 Jul	PMID: 24752257
55.	Asia/India	NDM-1/*E. coli*	1	2014 Apr	PMID: 24739981
56.	Asia/India	NDM-1/*K. Pneumonia*	6	2014 Jan-Mar	PMID: 24739834
57.	Asia/Kolkata, India	NDM-1/*Klebsiella pneumoniae, Enterobacter cloacae*	2	2014 Mar	PMID: 24336426
58.	Asia/Kolkata, India	*NDM-1/Vibrio fluvialis*	27	2016 Oct	PMID: 27649032
59.	Asia/Maharashtra, India	NDM-10/*Klebsiella pneumoniae*	1	2016 Jan	PMID: 26776144
60.	Asia/Pune, India	*NDM-1(*352*), NDM-5(*97*), NDM-4(*28*), NDM-7(*28*)/E. coli*	510	2016 Sep	PMID: 27600040
61.	Asia/Kolkata	NDM-1/*Vibrio fluvialis*	27	2016 Oct	PMID: 27649032
62.	Asia/Middle East+	NDM-5*/ Escherichia coli*	1	2016 May	PMID: 27217442
63.	Asia/Azerbaijan/Iran	*NDM-1/Enterobacteriaceae*	7	2016 Nov	PMID: 27655293
64.	Asia/Japan	NDM-7/ *Escherichia coli*	1	2014 Dec	PMID: 25193039
65.	Asia/Japan	NDM-3/*Escherichia coli*	1	2014 Jun	PMID: 24687501
66.	Asia/Japan	NDM-4, NDM-5*/ Klebsiella pneumoniae*	2	2016 Jun	PMID: 27185797
67.	Asia/Daejeon, Korea	NDM-1/ *Acinetobacter pittii*	2	2015 Sep	PMID: 26206691
68.	Asia/South Korea	NDM-5/*Klebsiella pneumonia*	1	2015 Jun	PMID: 25988911
69.	Asia/South Korea	*NDM-5/ Escherichia coli*	3	2016 Jun	PMID: 27049587
70.	Asia/Korea	NDM-1*/ Klebsiella pneumoniae*	2	2016 Jan	PMID: 26824953
71.	Asia/Korea	NDM-1/ *E coli*	5	2016 Feb	PMID: 26653860
72.	Asia/South Korea	NDM-9/*Klebsiella variicola*	3	2017 Jan	PMID: 28087584
73.	Asia/Lebanon	NDM-1/ *Acinetobacter baumannii*	4	2014 Apr	PMID: 24560830
74.	Asia/Lebanon	NDM-1*/ Acinetobacter pittii*	1	2016 Apr	PMID: 27222717

75.	Asia/Kathmandu, Nepal	NDM-13/*Escherichia coli*	1	2015 Sep	PMID: 26169399
76.	Asia/Nepal	NDM-12/ *E coli*	1	2014 Oct	PMID: 25092693
77.	Asia/Nepal	NDM-1/*Providencia rettgeri*	3	2014 Feb	PMID: 24484534
78.	Asia/Pakistan	NDM-1/*Klebsiella pneumoniae (8), E. coli (6), Enterobacter cloacae (2), Enterobacter aerogenes (5), Citrobacter freundii (4), Acinetobacter iwoffi (1), Providencia sp. (2)*	28	2015 Jun	PMID: 25988236
79.	Asia/Pakistan	NDM-1/*NA*	12	2015 Apr	PMID: 25764102
80.	Asia/Pakistan	NDM-1/*Salmonella enterica serovarAgona*	2	2015 Jan	PMID: 25378577
81.	Asia/Pakistan	NDM-1/*Escherichia coli (30), Enterobacter cloacae (21), Citrobacter freundii (4), Acinetobacter baumannii (3), Klebsiella pneumoniae (3), Pseudocitrobacter faecalis (2), Providencia rettgeri (2), Citrobacter braakii (1)*	66	2014 Sep	PMID: 24982081
82.	Asia/Philippines	NDM-1/ *Klebsiella pneumoniae*<br>NDM-7/ *Klebsiella pneumoniae*	1<br>1	2016 Mar	PMID: 27032000
83.	Asia/Singapore	NDM-1/ *Enterobacter cloacae*	4	2015 Oct	PMID: 26454748
84.	Asia/Singapore	NDM-1/*Klebsiellapneumonia*	2	2015 Aug	PMID: 26308279
85.	Asia/Singapore	NDM-1/*Escherichia coli*	1	2013 Dec	PMID: 24356827
86.	Asia/Singapore	NDM-1/*Enterobacter cloacae*	6	2016 Feb	PMID: 26454748
87.	Asia/Malaysia	NDM-1/*Acinetobacter pittii*	1	2016 Feb	PMID: 26742728
88.	Asia/Malaysia	NDM-1/*Klebsiellapneumoniae, E coli,Klebsiella ornithinolytica*	6318	2015 Dec	PMID: 26712667
89.	Asia/Taiwan	NDM-1/*Klebsiella pneumoniae, E coli*	2	2014 Aug	PMID: 25144712
90.	Asia/Taiwan	NDM-1/*E. coli*	1	2015 Apr	PMID: 25074627
91.	Asia/Taiwan	NDM-1/*Acinetobacter*	1	2014 Oct	PMID: 25059771
92.	Asia/Taiwan	NDM-1/*Escherichia coli*	1	2013 Dec	PMID: 24354657
93.	Asia/Thailand	NDM-1/*Klebsiella pneumoniae (2), E. coli (1)*	3	2014 Nov	PMID: 25096073
94.	Asia/Gaziantep, Turkey	NDM-1/*K pneumoniae (3), E. cloacae (1), S. marcescens (1), K. oxytoca (1)*	6	2015 May	PMID: 26051720
95.	Asia/Istanbul, Turkey	NDM-1/*K. pneumoniae (4), E. cloacae (8)*	12	2014 May	PMID: 24550328
96.	Asia/Istanbul,Turkey	NDM-1/*Klebsiella pneumoniae*	7	2015 Nov	PMID: 26354347<br>PMID: 26860360

97.	Asia/Istanbul,Turkey	NDM-1 *Klebsiella pneumoniae*	8	2016 Feb	
98.	Asia/Vietnam	NDM-1/*Klebsiella pneumoniae (22), Enterobacter cloacae (20), E. coli (15), Citrobacter freundii (9), K. oxytoca (1), E. aerogenes (1) and Providencia rettgeri (1).*	45	2015 Jun	PMID: 25732142
99.	Asia/Southern Vietnam	NDM-1/*Vibrio cholera*	1	2015 May	PMID: 25683557
100.	Asia/Vietnam	NDM-1/ *Acinetobacter baumannii*	2	2015 Oct	PMID: 26471294
101	Asia/Vietnam	NDM-1/ *Acinetobacter baumannii*	23	2016 Oct	PMID: 27714593
102.	Asia/Vietnam	*NDM-1/Acinetobacter calcoaceticus-baumannii*	1	2017 Feb	PMID: 27714593
103.	Asia/ Slovakia	NDM-1/*Klebsiella pneumoniae*	1	2014 Sep	PMID: 25702288
104.	Asia/Slovakia	NDM-1/*P. aeruginosa*	6	2015 Feb	PMID: 25343711
105.	Asia/Saudi Arabia	NDM-1/ *Acinetobacter baumannii*	3	2016 May	PMID: 27183378
106.	Asia/Arabian Peninsula	NDM-7/ *Escherichia coli*	157	2017 Feb	PMID: 28156193
107.	Asia/Yemen	NDM-1/*Klebsiella pneumoniae (8) Enterobacter cloacae (2)*	10	2014 Oct	PMID: 25009193
108.	Africa/North Africa, Algiers	NDM-1/*Acinetobacter baumannii*	10	2015 Jun	PMID: 26194827
109.	Africa/Algeria	NDM-1/*Acinetobacter baumannii*	11	2014 Nov	PMID: 25240726
110.	Africa/Algeria	NDM-5/*Escherichia coli*	3	2014 Sep	PMID: 24982080
111.	Africa/Algeria	NDM-1/*Acinetobacter baumannii*	32	2016 Jan	PMID: 26615460
112.	Africa/Algeria	NDM-5/ *E. coli* NDM-5/ *E. coli*	1	2016 Jan	PMID: 26741510
113.	Africa/Algeria	NDM-5/ *Escherichia coli*	3	2015 Sep	PMID: 26566444
114.	Africa/Algeria	NDM-1/*A. baumannii (4), A. nosocomialis (1)*	1	2016 Jun	PMID: 26741510
115.	Africa/Algeria		5	2016 Dec	PMID: 28007519
116.	Africa/Algeria	NDM-1/*Acinetobacter baumannii*	1	2016 Dec	PMID: 27835841
117.	Africa/Greater Johannesburg Area	NDM-1/*Klebsiella pneumoniae (28), Enterobacter cloacae (5), Klebsiella oxytoca (2), Serratia marcescens (2), Citrobacter amalonaticus (1)*	38	2015 Apr	PMID: 25909482
118.	Africa/KwaZulu-Natal,	NDM-1/*Enterobacter cloacae (2),*	4	2014 Jul	PMID: 24853768

	South Africa	*Citrobacter freundii (1), Serratia marcescens (1)*			
119.	Africa/Libya	NDM-1/*Acinetobacter baumannii, Pseudomonas aeruginosa, Pseudomonas putida, Escherichia coli, Klebsiella pneumoniae, Enterobacter gergoviae*	8	2015 Aug	PMID: 26294290
120.	Africa/Libya	NDM-1/*Acinetobacter baumannii*	8	2016 Apr	PMID: 27216382
121.	Africa/Madagascar	NDM-1/*Klebsiella pneumoniae*	1	2015 Jun	PMID: 25845871
122.	Africa/Egypt	NDM-1/*Klebsiella pneumoniae*	8	2015 Dec	PMID: 26686939
123.	Africa/Egypt/Cairo	*NDM-1/NA*	24	2016 Sep	PMID: 27685673
124.	Africa/Egypt	NDM-1/*K. pneumoniae, E. coli, P.aeruginosa, A. baumannii*	24	2016 Dec	PMID: 27685673
125.	Africa/Tunisia	NDM-1/*Klebsiella pneumoniae*	1	2016 Sep	PMID: 27659734
126.	Africa/Tunisia	NDM-1/*A. baumannii*	1	2017 Jan	PMID: 28099062
127.	Africa/Tunisia	*NDM-1/ K. pneumoniae*	6	2016 Nov	PMID: 27965626
128.	Africa/Tunisia	NDM-1/ *K. pneumoniae*	1	2017 Jan	PMID: 27659734
129.	America/Riode Janeiro, Brazil	NDM-1/*Enterobacter hormaechei*	1	2015 Apr	PMID: 25473727
130.	America/Argentina	NDM-1/*Acinetobacter bereziniae*	1	2016 Mar	PMID: 26966220
131.	America/Brazil	NDM-1/*P. rettgeri (1), Enterobacter cloacae (3)*	4	2015 Feb	PMID: 25466163
132.	America/Brazil	NDM-1/*Acinetobacter baumannii*	1	2014 Dec	PMID: 25288087
133.	America/Porto Alegre, Brazil	NDM-1/*Enterobactercloacae (9), Morganella morganii (2)*	11	2014 Aug	PMID: 24857802
134.	America/Brazil	NDM-1/*Enterobacter hormaechei*	6	2014 Apr	PMID: 24449772
135.	America/Brazil	NDM-1/*Providencia rettgeri*	1	2015 Jun	PMID: 25989100
136.	America/California	NDM-1/ *K. pneumoniae*	4	2015 May	PMID: 25977423
137.	America/Colorado	NDM-1/ *K. pneumoniae*	8	2014 Apr	PMID: 24602944
138.	America/Colorado	NDM-1/*NA*	5	2014 Apr	PMID: 24602952
139.	America/Colorado	NDM-1/ *K. pneumoniae*	6	2016 Dec	PMID: 27977640
140.	America/Florida	NDM-1/*Klebsiellapneumonia*	1	2016 Apr	PMID: 26983001

141.	America/Ecuador	NDM-1/*Providencia rettgeri*	1	2015 Dec	PMID: 2784289
142.	America/Atlanta, Georgia	NDM-1/*Escherichia coli*	1	2015 Mar	PMID: 25744985
143.	America/Illinois	NDM-1/ *Escherichia coli*	39	2014 Oct	PMID: 25291580
144.	America/Jamaica	NDM-1/ *Klebsiella pneumonia*	1	2016 Feb	PMID: 26927461
145.	America/Mexico City	NDM-1/*Escherichia coli (1), Enterobacter cloacae (1), Klebsiella pneumoniae (3)*	5	2015 Nov	PMID: 26282410
146.	America/Mexico	NDM-1/*Klebsiella pneumoniae*	1	2014 Mar	PMID: 24569387
147.	America/Paraguay	NDM-1/*Acinetobacter pittii*	2	2014 Sep	PMID: 24793901
148.	America/Pennsylvania	NDM-1/*K. pneumoniae, Escherichia coli*	2	2014 Jan	PMID: 24377764
149.	America/Uruguay	NDM-1/*Morganella morganii*	1	2015 Jan	PMID: 25447717
150.	Europe/Azerbaijan	*NDM-1/NA*	7	2016/Sep	PMID: 27655293
151.	Europe/Bulgaria	NDM-1/*Escherichia coli*	12	2014 Apr	PMID: 24514099
152.	Europe/Denmark	NDM-7*Klebsiella pneumoniae*	1		
		NDM-5/ *Escherichia coli*	1	2015 Nov	PMID: 26307465
153.	Europe/Bulgaria	NDM-1/*Klebsiella pneumoniae*	2	2016 Jan	PMID: 26817486
154.	Europe/Bulagria	NDM-1/ *Klebsiella pneumoniae*	1	2015 Sep	PMID: 26594376
155.	Europe/Ukraine	NDM-1/*Klebsiella pneumoniae*	1	2016 Apr	PMID: 27129373
156.	Europe/France	NDM-1/*Proteus mirabilis*	1	2015 Jan	PMID: 25239462
157.	Europe/France	NDM-1/*Morganella morganii*	1	2014 Oct	PMID: 25081858
158.	Europe/France	*NDM-7/ Escherichia coli*	1	2016 Jun	PMID: 27178253
159.	Europe/France	NDM-1/*Acinetobacter pittii*	1	2016 Dec	PMID: 27999060
160.	Europe/Germany	NDM-1/*S. marcescens*	1	2015 Apr	PMID: 25468904
161.	Europe/Germany	NDM-1/ *Klebsiella pneumoniae (1), Escherichia coli (1)*	2	2016 May	PMID: 27237423
162.	Europe/Greece	NDM-1/ *K. pneumoniae*	78	2014 Aug	PMID: 24739146
163.	Europe/Greece	NDM-1/*Acinetobacter baumannii*	1	2016 Dec	PMID: 27773496
164.	Europe/Italy	NDM-1/*Klebsiellapneumonia*	1	2015 Jun	PMID: 26116560
165.	Europe/Italy	NDM-4/*Escherichia coli*	8	2014 Jun	PMID: 24906230
166.	Europe/Italy	NDM-5/ *Escherichia coli*	1	2017 Jan	PMID: 28159670
167.	Europe/Ireland	*NDM-1/ Klebsiella pneumoniae (9), Escherichia coli (1)*	10	2016 Dec	PMID: 27624807
168.	Europe/Croatia	NDM-1/*Citrobacterkoseri*	1	2015 oct	PMID: 26484384
169.	Europe/Croatia	NDM-1/ *Klebsiella pneumoniae*	7	2016 May	PMID: 27174090
170.	Europe/Poland	NDM-1/*Klebsiella pneumoniae*	374	2015 Sep	PMID: 26386745
171.	Europe/Poland	NDM-1/*Klebsiella pneumoniae, Escherichia coli*	2	2015 Jun	PMID: 26084313

172.	Europe/Poland	NDM-1/*Klebsiella pneumoniae*	1	2014 Sep	PMID: 25242796
173.	Europe/Romania	NDM-1/*E. cloacae*	2	2014 Mar	PMID: 24346066
174.	Europe/Romania	NDM-1/*Klebsiella pneumoniae*	8	2015 Nov	PMID: 26599338
175.	Europe/Serbia	NDM-1/*Pseudomonas aeruginosa*	6	2014 Mar	PMID: 24343100
176.	Europe/Serbia	*NDM-1/ Escherichia coli*	1	2016 Apr	PMID: 27074434
177.	Europe/Turkey	NDM-1/*Acinetobacter baumannii*	2	2015 Sep	PMID: 26296677
178.	Europe/Turkey	NDM-1/*Acinetobacter pittii*	1	2014 Dec	PMID:25096072
179.	Europe/Turkey	NDM-1/*Klebsiella pneumoniae*	4	2016 Sep	PMID: 27942358
180.	Europe/Turkey	NDM-1/*Klebsiella pneumoniae*	2	2016 Dec	PMID: 27939808
181	Europe/London	NDM-1/*Klebsiella spp. (180), Escherichia coli (80)*	326	2014 Jul	PMID: 24769387
182.	Australia/Brisbane	NDM-1/*Klebsiella pneumoniae*	2	2015 Mar	PMID: 25758700
183.	Australia/Perth	NDM-1/*Enterobacter cloacae*	1	2014 Apr	PMID: 24794661
184.	Australia/New Zealand	NDM-5/ *Klebsiella pneumoniae*	1	2016 Dec	PMID: 27999016
